# Hydroxychloroquine sulfate for IgA nephropathy: mechanisms and therapeutic potential in improving proteinuria and alleviating disease progression - a literature review

**DOI:** 10.1186/s12882-025-04262-5

**Published:** 2025-07-01

**Authors:** Liao Yilei, Du Yating, Fang Yaxuan, Liu Chenxuan, Cheng Tingzhu, Li Jinpu, Rao Xiangrong, Guo Chuan

**Affiliations:** Guang’anmen Hospital, China Academy of Chinese Medical Sciences, Beijing, 100053 China

**Keywords:** IgA nephropathy, Proteinuria, Hydroxychloroquine, Galactose-deficient IgA1, Mechanism

## Abstract

IgA nephropathy (IgAN), the most common form of glomerulonephritis worldwide, often progresses to chronic kidney failure within 10 to 15 years. Despite its clinical importance, effective disease-modifying therapies for IgAN remain limited. Proteinuria is well recognized as both a prognostic biomarker and a modifiable therapeutic target in IgAN. Several randomized controlled trials conducted among Chinese patients with IgAN have demonstrated the efficacy of hydroxychloroquine (HCQ) in reducing proteinuria. The Kidney Disease: Improving Global Outcomes (KDIGO) guidelines also suggest that HCQ may exert potential therapeutic effects in IgAN. However, the molecular mechanisms underlying the renoprotective effects of HCQ remain incompletely understood. This review synthesises current evidence on HCQ’s therapeutic mechanisms in IgAN, highlighting its multifaceted roles in: (1) suppressing pathogenic galactose-deficient IgA1 synthesis through modulation of mucosal immunity, Toll-like receptor (TLR) signaling, IL-6 pathways, and complement activation; (2) inhibiting autophagy-mediated antigen presentation via major histocompatibility complex class II (MHC-II) molecules; (3) modulating non-canonical autophagy pathways to attenuate human mesangial cells (HMCs) proliferation and protect podocytes; and (4) demonstrating antithrombotic effects. Collectively, HCQ demonstrates multifaceted mechanisms for proteinuria reduction in IgAN while maintaining a favorable safety profile.

## Clinical trial number

Not applicable.

## Background

IgA nephropathy (IgAN) is the most prevalent form of glomerulonephritis worldwide and a major contributor to end-stage renal disease (ESRD). The median age at diagnosis typically ranges from 40 to 45 years, with cases frequently identified at younger ages [1]. In China, IgAN accounts for 30–54% of all glomerulonephritis cases and represents the most common primary glomerular disease diagnosed by renal biopsy [2–4].

IgAN primarily affects the glomeruli and is pathologically characterised by mesangial deposition of IgA-dominant immune complexes (ICs). These deposits induce proliferation of human mesangial cells (HMCs) and expansion of the mesangial matrix [5]. Clinically, patients typically present with episodic gross hematuria, asymptomatic microscopic hematuria, and/or varying degrees of proteinuria [6].

Proteinuria represents a critical clinical manifestation of IgAN and has been established as an independent risk factor for renal function deterioration [7, 8]. A large-scale cohort study of IgAN patients demonstrated that the risk of the composite kidney outcome began to increase at time-averaged proteinuria (TAP) of ≥ 0.5 g/day. The risk rose 8.5-fold at TAP > 1.0 g/day and 38-fold at TAP > 2.0 g/day compared to patients with TAP < 0.3 g/day, demonstrating a strong dose-response relationship [9]. Moreover, proteinuria has been validated as a reliable surrogate endpoint for assessing treatment efficacy and predicting progression to ESRD in IgAN [10, 11].

Hydroxychloroquine (HCQ), a 4-aminoquinoline derivative structurally similar to chloroquine (CQ) but with β-hydroxylation modification [12], has emerged as a promising therapy for IgAN [13–15]. HCQ’s established pharmacokinetics (hepatic metabolism, fecal excretion) [12] and decades of use in systemic lupus erythematosus (SLE)/ lupus nephritis (LN) support its repurposing potential [16–18].

Clinical trials, primarily conducted in Chinese populations, have consistently demonstrated the efficacy and favorable safety profile of HCQ in reducing proteinuria (Table 1) [14, 15, 19–27]. The proposed mechanisms of action include inhibition of Toll-like receptor (TLR) signaling pathways, suppression of galactose-deficient IgA1 (Gd-IgA1) production, and attenuation of intrarenal inflammation [27]. The Kidney Disease: Improving Global Outcomes (KDIGO) guidelines suggest that HCQ may have therapeutic potential in IgAN. While current data are encouraging, validation in diverse ethnic groups is needed [28]. Two forthcoming multicenter randomized controlled trials - the Chinese HOT-IgAN study comparing HCQ with Tripterygium wilfordii and the European QUIgAN trial evaluating eGFR preservation - will provide high-level evidence for HCQ’s use in IgAN [29].

However, the precise mechanisms underlying HCQ’s renoprotective effects remain incompletely understood, likely reflecting gaps in our fundamental knowledge of IgAN pathogenesis. This review systematically examines potential mechanisms by which HCQ may ameliorate proteinuria and mitigate IgAN.

**Table 1:** Efficacy and safety of hydroxychloroquine in IgA nephropathy: clinical trial evidence

Reference	Sample size	Duration	Drug dosage	24 h-uTP, g/d	eGFR, ml/min/1.73m^2^	Effects	Adverse effects
He, 2024^19^	159 IgAN patients: • 57 HCQ + RAASi • 52 LEF + RAASi 50 RAASi	6 months	0.2 g twice a day (eGFR > 60 ml/min/1.73m^2^) 0.1 g 3 times a day (eGFR 45–59 ml/min/1.73m^2^) 0.1 g twice a day (eGFR 30–44 ml/min/1.73m^2^)	HCQ + RAASi: 0.89 (0.70, 1.23) to 0.22 (0.14, 0.49), *P* < 0.001; LEF + RAASi: 0.84 (0.70, 1.07) to 0.37 (0.22, 0.64), *P* < 0.001; RAASi: 0.77 (0.67, 0.96) to 0.47 (0.36, 0.69), *P* < 0.001	HCQ + RAASi: 74.83 ± 14.14 to 77.39 ± 13.48 LEF + RAASi: 79.86 ± 10.19 to 82.29 ± 9.83 RAASi: 79.26 ± 17.22 to 80.65 ± 17.03	HCQ combined with RAASi shows greater proteinuria reduction and renal function preservation than RAASi alone.	1. Three patients in the HCQ group experienced elevated transaminase levels, but their levels returned to normal after receiving liver protection treatment. 2. SAEs.
Liu, 2022^15^	425 IgAN patients: • 183 RAASi • 59 HCQ + RAASi • 145 RAASi + P ± IM^a^ 38 HCQ + RAASi + P ± IM^a^	6 months	0.2 g twice a day (eGFR > 45 ml/min/1.73m^2^) 0.1 g twice or 3 times a day (eGFR 30–45 ml/min/1.73m^2^) 0.1 g once a day (eGFR < 30 ml/min/1.73m^2^)	RAASi: 0.7 (0.5, 0.9) to 0.46 (0.27, 0.89), *P* > 0.05; HCQ + RAASi: 0.8 (0.6, 1.2) to 0.61 (0.40, 0.97), *P* < 0.05; RAASi + P ± IM: 1.06 (0.54, 2.11) to 0.65 (0.31, 1.35), *P* < 0.05 HCQ + RAASi + P ± IM: 1.29 (1.02, 2.18) to 0.89 (0.41, 1.27), *P* < 0.05	RAASi: 97.4 (77.3, 119.9) to 96.06 (61.33, 118.92); HCQ + RAASi: 91.1 (63.3, 119.5) to 84.78 (55.85, 121.77); RAASi + P ± IM: 76.2 (60.0, 102.2) to 85.92 (65.36, 121.85) HCQ + RAASi + P ± IM: 74.6 (51.0, 107.1) to 62.95 (41.10, 99.58)	HCQ adjunct therapy reduces residual proteinuria in 86.2% of IgAN patients previously treated with supportive care plus immunosuppressants (P/IM).	None
Shi, 2025^20^	49 IgAN patients	6 months	0.2 g twice a day (eGFR > 60 ml/min/1.73m^2^) 0.1 g 3 times a day (eGFR 45–59 ml/min/1.73m^2^) 0.1 g twice a day (eGFR 30–44 ml/min/1.73m^2^)	921 (676, 1,580) to 514 (287, 861)	105.0 ± 15.0 to 103.7 ± 13.9	The blood concentrations of HCQ and its metabolite DCQ were significantly positively correlated with the reduction rate of proteinuria in patients with IgAN and efficacy was significantly correlated with HCQ	1. Two patients experienced nausea. 2. No SAEs.
Tang, 2022^21^	180 IgAN patients	24 months	0.2 g twice a day (eGFR > 60 ml/min/1.73m^2^) 0.1 g 3 times a day (eGFR 45–59 ml/min/1.73m^2^) 0.1 g twice a day (eGFR 30–44 ml/min/1.73m^2^) 0.1 g once a day (eGFR < 30 ml/min/1.73m^2^)	1.69 (1.24, 2.30) to 1.00 (0.59, 1.60), *P* < 0.001	65.82 ± 25.22 to 62.15 ± 25.81, *P* = 0.003	1. HCQ safely reduces proteinuria and preserves renal function across all eGFR stages in IgAN patients. 2. Patients with different eGFR levels respond differently to HCQ.	None
Yang, 2024^22^	73 IgAN patients: • 38 HCQ + RAASi 35 RAASi	6 months	0.2 g twice a day (eGFR > 60 ml/min/1.73m^2^) 0.1 g 3 times a day (eGFR 45–59 ml/min/1.73m^2^) 0.1 g twice a day (eGFR 30–44 ml/min/1.73m^2^) 0.1 g once a day (eGFR < 30 ml/min/1.73m^2^)	HCQ + RAASi: 0.832 (0.701–1.395) to 0.542 (0.355–0.815) RAASi: 0.943 (0.685–1.726) to 0.884 (0.499–1.001)	HCQ + RAASi: 100.60 ± 26.19 to 95.79 ± 27.07 RAASi: Not mentioned	HCQ safely reduces proteinuria in Chinese patients with IgAN, with efficacy showing concentration-dependent effects.	1. One patient experienced mild renal impairment. 2. No SAEs.
Gao, 2017^23^	28 IgAN patients: • 14 HCQ and Losartan 14 Losartan	24 weeks	0.1 g twice a day	HCQ + Losartan: 0.90 ± 0.45 to 0.54 ± 0.23 Losartan: 0.78 ± 0.25 to 0.74 ± 0.31	HCQ + Losartan: 83 ± 18 to 81 ± 21 Losartan: 84 ± 19 to 81 ± 19	The treatment with HCQ combined with losartan significantly reduced proteinuria in patients with IgAN (Proteinuria had partial or complete remission in 42.9% of the HCQ group, versus only 14.3% in the controls).	1. One patient developed drug-induced rash and recovered rapidly after discontinuing the drug. 2. No SAEs.
Yang, 2018^14^	180 IgAN patients: • 90 HCQ + RAASi 90 RAASi	6 months	0.2 g twice a day (eGFR > 45 ml/min/1.73m^2^) 0.1 g twice or 3 times a day (eGFR 30–45 ml/min/1.73m^2^) 0.1 g once a day (eGFR 15–30 ml/min/1.73m^2^)	HCQ + RAASi: 1.5 (1.2, 2.1) to 0.8 (0.7, 1.2) RAASi: 1.5 (1.2, 1.9) to 1.2 (0.8, 1.8)	HCQ + RAASi: 51.2 ± 21.7 to 47.7 ± 25.2 RAASi: 51.2 ± 21.7 to 50.5 ± 17.6	HCQ combined with RAASi safely achieved superior proteinuria reduction (> 30%) in IgAN patients compared to RAASi alone (70% vs. 45.6% at 6 months; *p* < 0.01).	1. Two patients were allergic to HCQ. One patient had mild liver function abnormalities (ALT 155 IU/L, AST 86 IU/L [15–40]). The liver function returned to normal three months after the drug withdrawal. One patient experienced palpitations, and one patient had an increase in intraocular pressure. Three patients had isolated skin reactions, including rash, pruritus, alopecia, or desquamation. These symptoms resolved after the drug withdrawal. 2. No SAEs.
Liu, 2019^27^	60 IgAN patients: • 30 HCQ 30 Placebo	6 months	0.2 g twice a day (eGFR > 60 ml/min/1.73m^2^) 0.1 g 3 times a day (eGFR 45–59 ml/min/1.73m^2^) 0.1 g twice a day (eGFR 30–44 ml/min/1.73m^2^) Not take (eGFR < 30 ml/min/1.73m^2^)	HCQ: 1.6 (1.1, 2.2) to 0.9 (0.6, 1.0) Placebo: 1.9 (1.3, 2.6) to 1.9 (0.9, 2.6)	HCQ: 52.1 ± 19.7 (baseline) Change of eGFR 4.5% (− 12.3%, 23.1%) Placebo: 55.5 ± 18.7 (baseline) Change of eGFR 0.0% (− 12.6%, 19.3%)	HCQ treatment resulted in both a significantly greater percentage reduction and lower absolute proteinuria levels compared to placebo at 6 months. HCQ in addition to optimized RAASi significantly reduced proteinuria in patients with IgAN over 6 months	1. Two patients experienced a decline in eGFR: one patient had a 29.0% decrease in eGFR at 4 months, and the other patient had a 33.4% decrease in eGFR at 6 months. One patient was allergic to HCQ. The symptoms subsided after the drug was discontinued. One patient experienced occasional dizziness and pruritus, and the problem was resolved after reducing the dosage. One patient developed skin pigmentation, transient palpitations, and nausea, and the symptoms spontaneously resolved. 2. No SAEs.
Tang, 2020^24^	52 IgAN patients: • 26 HCQ + IS^b^ 26 IS^b^	6 months	0.2 g twice a day (eGFR > 60 ml/min/1.73m^2^) 0.1 g twice or 3 times a day (eGFR 30–59 ml/min/1.73m^2^) 0.1 g once a day (eGFR 15–30 ml/min/1.73m^2^)	HCQ + IS: 2.35 (1.47, 2.98) to 1.10 (0.85, 1.61) IS: 2.35 (1.54, 2.98) to 1.24 (0.87, 2.58)	HCQ + IS: 47.65 (34.65, 67.48) to 50.91 (30.96, 71.14) IS: 47.65 (34.65, 67.48) to 56.39 (19.37, 79.24)	Use of HCQ achieved a similar reduction in proteinuria compared to conventional IS therapy in patients with IgAN who had insufficient responses to IS therapy.	1. One patient stopped taking HCQ due to nausea and diarrhea. One patient developed skin pigmentation. 2. No SAEs.
Yang, 2019^25^	184 IgAN patients: • 92 HCQ + RAASi 92 P	6 months	0.2 g twice a day (eGFR > 45 ml/min/1.73m^2^) 0.1 g twice or 3 times a day (eGFR 30–45 ml/min/1.73m^2^) 0.1 g once a day (eGFR 15–30 ml/min/1.73m^2^)	HCQ + RAASi: 1.7 (1.2, 2.3) to 0.8 (0.6, 1.1) P: 1.8 (1.3, 2.5) to 0.7 (0.3, 1.1)	HCQ + RAASi: 56.8 ± 20.4 to 56.8 ± 20.4 P: 55.2 ± 22.9 to 55.2 ± 22.9	HCQ showed modestly less proteinuria reduction but superior safety than corticosteroids in IgAN.	1. One patient experienced a decrease in eGFR (29.0% and 33.4%), leading to adjustments in the treatment. Other adverse reactions included palpitations (1 case), nausea (1 case), abdominal distension (1 case), pruritus (2 cases), skin pigmentation (1 case), alopecia (1 case), and rash (1 case). 2. No SAEs.
Si, 2023^26^	78 IgAN patients: • 39 HCQ 39 CS	24 months	0.2 g twice a day (eGFR > 60 ml/min/1.73m^2^) 0.1 g twice or 3 times a day (eGFR 30–59 ml/min/1.73m^2^) 0.1 g once a day (eGFR 15–30 ml/min/1.73m^2^)	HCQ: 1.72 (1.44, 2.35) to 0.97 (0.51, 1.37) CS: 1.86 (1.33, 2.55) to 0.53 (0.25, 1.81)	HCQ: 68.54 ± 24.86 to 66.10 ± 28.00 CS: 68.37 ± 21.02 to 67.04 ± 23.80	HCQ shows durable antiproteinuric efficacy in IgAN with 24-month renal protection and better safety, achieving long-term outcomes comparable to corticosteroids despite slightly weaker initial effects	1. Nausea (2 cases), skin pigmentation (2 cases), palpitations (1 case). 2. No SAEs.

HCQ, Hydroxychloroquine; IgAN, IgA nephropathy; LEF, leflunomide; SAEs, severe adverse events; RAASi: renin-angiotensin-aldosterone system inhibitors; P/CS, corticosteroids; IM/ IS, immunosuppressives; eGFR, estimated glomerular filtration rate; DCQ, desethylchloroquine

a IM includes cyclophosphamide, azathioprine, cyclosporine, tacrolimus, mycophenolate mofetil, cyclosporine A, leflunomide, tripterygium glycosides, etc

b IS includes cyclophosphamide, mycophenolate mofetil, tacrolimus, Cyclosporine A, and leflunomide

## HCQ mitigates mucosal immune response and inhibits Gd-IgA1 synthesis

### “Four-hit” hypothesis and pathogenic Gd-IgA1 synthesis

**Fig. 1:**
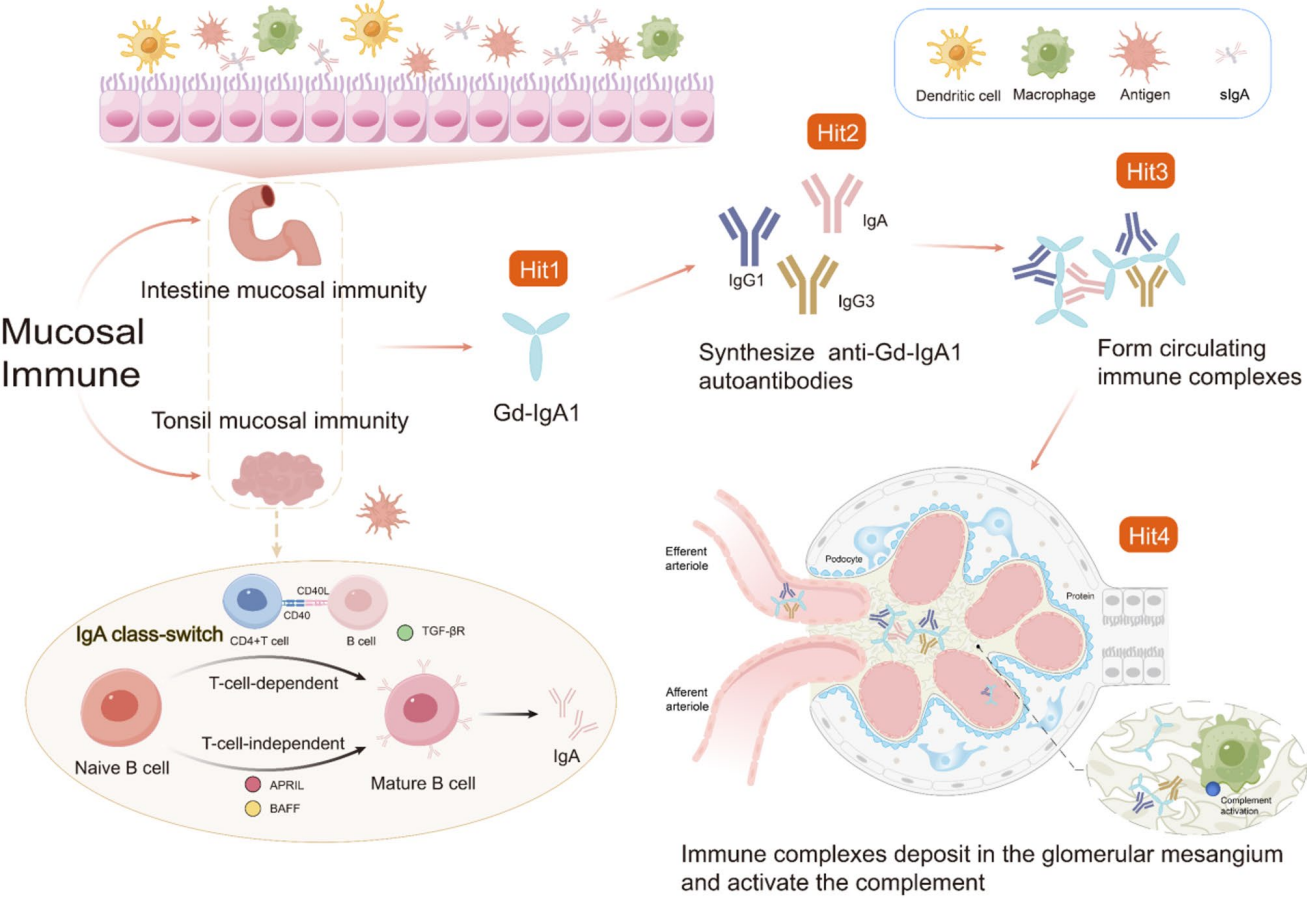
“Four-hit” hypothesis and mucosal immunity. Antigens stimulate mucosal immunity, including the tonsils and intestinal mucosa. Naïve B cells encounter antigens in the inductive sites of mucosal immunity. IgA-specific CSR occurs via T-cell-dependent and T-cell-independent pathways. In the T-cell-dependent pathway, CSR is induced by TGF-β1, IL-6, and IL-10. CD40 on B cells binds to CD40L on activated CD4 + T cells, providing a costimulatory signal for B cell activation and IgA CSR. In the T-cell-independent pathway, CSR is mediated directly by TGF-β, IL-6, IL-10, BAFF, and APRIL. In IgAN, the first hit involves elevated circulating levels of Gd-IgA1. The second hit is the production of anti-Gd-IgA1 autoantibodies. The third hit occurs when these autoantibodies bind Gd-IgA1, forming circulating ICs. These ICs deposit in the glomerular mesangium (fourth hit), activating HMCs and complement, ultimately leading to renal injury. IgAN, IgA nephropathy; CSR, class-switch recombination; BAFF, B cell activation factor; APRIL, a proliferation-inducing ligand; Gd-IgA1, galactose-deficient IgA1; ICs immune complexes; HMCs, human mesangial cells

The “four-hit” hypothesis remains the predominant model of IgAN pathogenesis [5] (Fig. 1), with Gd-IgA1 serving as a central mediator [30]. Aberrant glycosylation of IgA1 facilitates the formation of pathogenic ICs either through glycan binding or anti-glycan antibody interaction [31], with genetic factors influencing multiple pathogenic stages [32, 33].

Evidence from renal transplantation studies highlights the systemic nature of IgAN: mesangial deposits resolve in non-IgAN recipients but recur in IgAN patients receiving normal kidneys [34]. This finding supports the notion that disease manifestation depends on IgA1 glycosylation structure rather than quantity [35].

IgA is the most produced antibody in the body [36]. While bone marrow-derived IgA1 is monomeric and fully galactosylated, the galactose-deficient forms in IgAN kidneys closely mucosal-type dimeric IgA1 [37], suggesting a potential mucosal origin [38].

Elevated serum Gd-IgA1 levels predict poorer renal outcomes [39–41]while their reduction is correlated with improved proteinuria [42]. Mechanistically, Gd-IgA1’s stronger mesangial binding [43] may be attributed to molecular mimicry: its N-acetylgalactosamine (GalNAc) residues resemble pathogen epitopes [36, 37]potentially inducing cross-reactive antibodies that explain infection-associated flares [32, 36, 44–46].

### Mucosal immunization and IgAN

The pathogenesis of IgAN involves two primary lymphoid tissues: the nasopharynx-associated lymphoid tissue (NALT) and the gut-associated lymphoid tissue (GALT) [32]. In humans, the tonsillar germinal center and Peyer’s patches in the small intestine serve as key inductive sites for IgA class-switch recombination (CSR) in naïve B cells [32, 38, 47] (Fig. 1). Following antigen exposure, activated B cells migrate to the lamina propria of mucosal membranes, where they function as effector cells [48].

#### TLR9/7 in disease progression

**Fig. 2:**
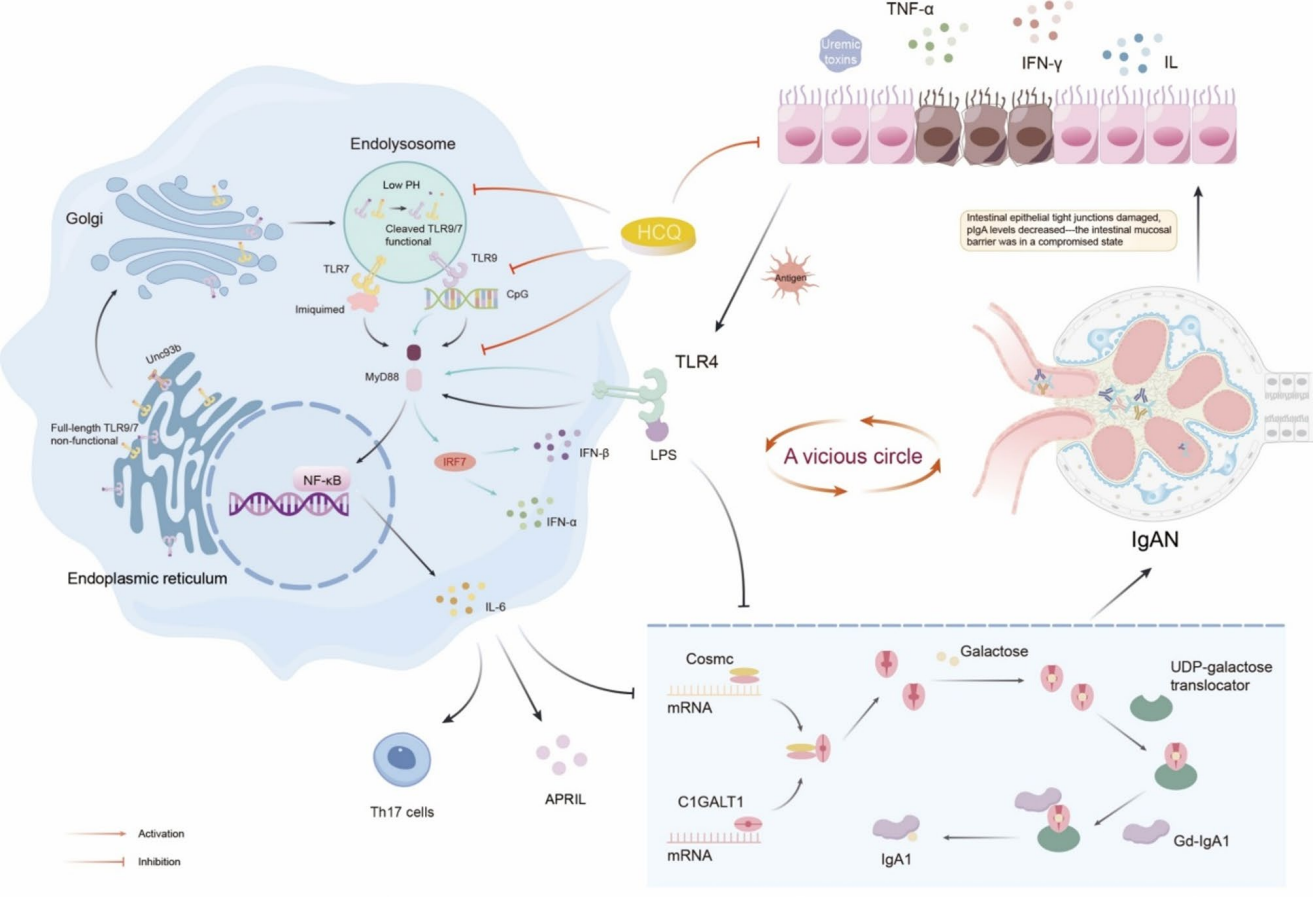
Mucosal Immunity in IgAN and the Role of HCQ. In patients with IgAN, accumulated uremic toxins promote the release of inflammatory substances, alter intestinal permeability, and impair intestinal epithelial tight junctions. Consequently, pathogens have an increased likelihood of crossing the intestinal mucosal barrier. LPS on the bacterial surface specifically activates the TLR4 signaling pathway, which generates IL-6, IFN-α, and IFN-β via the MyD88-NF-κB and MyD88-IRF7 pathways. Intracellular TLRs TLR9 and TLR7 are also present. The endoplasmic reticulum synthesizes intact TLR9/7 molecules. TLR9/7 exit the endoplasmic reticulum, traverse the Golgi apparatus, and reach the lysosome. In the lysosome, their ectodomains are cleaved and hydrolyzed, forming signaling-competent TLR9/7 molecules. Upon activation by ligands, TLR9 engages two signaling pathways, MyD88-NF-κB and MyD88-IRF7. In contrast, TLR7 activation leads to IL-6 production exclusively through the MyD88-NF-κB signaling pathway. IL-6 up-regulates APRIL, promotes Th17 cells production and inhibits the Cosmc gene expression. In the presence of Cosmc, C1GALT1 transfers β-galactose to 1,3-GalNAc via the UDP-galactose transporter, facilitating IgA1 formation. However, LPS and IL-6 inhibit Cosmc expression and decrease expression and activity of C1GALT1, decreasing the transfer of β-galactose to 1,3-GalNAc on IgA1. This results in an elevated level of Gd-IgA1, exacerbating the immune response and establishing a vicious cycle. HCQ repairs intestinal epithelial tight junctions, safeguards the junctional proteins ZO-1 and Occludin, and restricts pathogen translocation across the intestinal barrier. Additionally, HCQ inhibits lysosomal acidification, blocking the cleavage of the ectodomains of TLR9/7. It also prevents the binding of TLR9/7 to their ligands. At a concentration of 30 µM, HCQ completely suppresses the TLR-MyD88-NF-κB and TLR-MyD88-IRF7 signaling pathways. IgAN, IgA nephropathy; HCQ, hydroxychloroquine; Gd-IgA1, galactose-deficient IgA1; LPS, lipopolysaccharides; MyD88, myeloid differentiation factor 88; NF-κB, nuclear factor kappa-B; IRF7; interferon regulatory factor 7; Cosmc, core β1,3GalT-specific molecular Chaperone; C1GALT1, core-1 β1-3galactosyltransferase; TLR, Toll-like receptor; CpG-ODN, class c CpG oligodeoxynucleotides

Pathogenic microorganisms stimulate pattern recognition receptors (PRRs), including Toll-like receptors (TLRs), on B cells. Persistent TLR activation results in excessive production of Gd-IgA1 and O-glycan-specific antibodies [45]. Additionally, PRR activation increases secretion of B cell–activating factor (BAFF), further promoting IgA synthesis [32]. TLR signaling proceeds through the myeloid differentiation factor 88 (MyD88)-dependent and MyD88-independent pathways. TLR4, TLR7, and TLR9 activate the MyD88-dependent pathway, inducing IL-6 and type I interferon (IFN) production [49] (Fig. 2). In murine models, TLR9/7 activation triggers mesangial proliferation and IgA/IgG/C3 deposition [50, 51], accompanied by increased nitric oxide synthase (NOS) activity and the release of inflammatory mediators [50]. These responses contribute to local renal inflammation, altered glomerular permeability, tubulointerstitial injury, and proteinuria.

Notably, TLR activation upregulates IL-6 and a proliferation-inducing ligand (APRIL), further promoting IgA CSR and Gd-IgA1 production [50]. In vivo, pathogenic stimuli increase TLR9 and MyD88 expression in IgAN mice, exacerbating renal injury [52]. Clinical studies reveal elevated TLR expression in tonsils of IgAN patients, correlating with susceptibility to upper respiratory infections and disease progression [50]. Genetic analyses also link TLR9 polymorphisms to IgAN severity [52].

#### TLR4 in disease progression

Intestinal epithelial PRRs, particularly TLR4, uremic toxins, gut dysbiosis, and microbial flagellin-mediated TLR5 activation disrupt intestinal epithelial tight junctions [38, 53, 54]. Concurrently, a reduction in intestinal plasma cells impairs J-chain-dependent polymeric IgA (pIgA) assembly, impairing mucosal defense [46]. TLR4 recognizes bacterial lipopolysaccharides (LPS), inhibiting β-galactose transfer to GalNAc and promoting Gd-IgA1 formation [55, 56] (Fig. 2).

Notably, metabolomic profiling reveals that gut dysbiosis-associated depletion of short-chain fatty acids (SCFAs) and bile acids may directly promote aberrant IgA production [57], further linking intestinal homeostasis to IgAN development.

#### IL-6 in disease progression

IL-6 plays a central role in IgA1 galactosylation defects through multiple mechanisms (Fig. 2). First, it directly suppresses core β1,3GalT-specific molecular Chaperone (Cosmc) and core-1 β1-3galactosyltransferase (C1GALT1) expression, exacerbating galactose deficiency in IgA1 [56]. Second, IL-6 enhances terminal GalNAc sialylation, further impairing galactosylation [14]. In vitro, IL-6 is secreted following TLR9/TLR7-MyD88-NF-κB pathway activation. This cytokine upregulates APRIL, downregulates C1GALT1, and promotes the synthesis of Gd-IgA1 [50]. Clinically, elevated urinary IL-6 (> 2.5 ng/day) predicts a 7.8-fold higher risk of renal failure in IgAN patients, establishing it as an independent prognostic marker [58].

### The actions of HCQ in mucosal immune reactions of IgAN

HCQ demonstrates multiple mechanisms for attenuating mucosal immune activation in IgAN, including reduction of Gd-IgA1 production.

#### HCQ’s modulation of B cell function and TLR signaling

In vitro, Torigoe et al. revealed that CpG, a TLR9 ligand, stimulates B cell differentiation into CD27hiCD38hi plasmablasts with elevated TLR9 expression [59]. Among these, class-switched memory B cells produced the highest levels of IgG, contributing to inflammatory responses in infections and autoimmune diseases. HCQ effectively inhibited CpG-induced differentiation of these memory B cells into plasmablasts and suppressed subsequent IgG production. Mechanistically, HCQ inhibited CpG-induced phosphorylation of S6, an mTORC1 activator, and modulated intracellular metabolism, ultimately reducing B cell hyperactivity and antibody production.

Due to its weak alkalinity and lipophilicity, HCQ accumulates in acidic organelles [60]. Through pH-dependent diffusion and potential Na^+^/H^+^ exchanger-mediated uptake [61], HCQ concentrates up to 10,000-fold in endolysosomes [62]. This accumulation alkalinizes endolysosomal pH, impairing protein processing and degradation [62]. This alkalinization blocks TLR9/7 proteolytic cleavage, essential for downstream signaling [63].

An in vitro study by Lee et al. demonstrated that at 30µM HCQ concentration nearly completely suppressed both TLR9-MyD88-NF-κB/IRF7 and TLR7-MyD88-NF-κB pathways, reducing IL-6, IFN-α, and IFN-β production and inhibited the elevation of Gd-IgA1 [50] (Fig. 2). Notably, even at clinically relevant concentrations, HCQ maintains its inhibitory effect on TLR signaling without altering endosomal pH [64].

#### Anti-inflammatory effects on cytokines and renal protection

HCQ exerts its immunomodulatory effects through multiple mechanisms: (1) alkalinization of endolysosomes blocks proteolytic maturation of TLR9/7 and prevents ligand binding [17]; (2) HCQ more effectively suppresses IL-6 secretion and subsequent Gd-IgA1 and IgG-IgA immune complex formation compared to competitive TLR9/7 inhibitors like ODN2088 [50]; and (3) by reducing IL-6 production, HCQ rebalances the Th17/Treg ratio [65], thereby mitigating chronic inflammation and endothelial dysfunction [51].

Among TLR-induced cytokines, IL-6 directly promotes Gd-IgA1 production [56, 66–68]whereas type I IFNs (implicated in SLE/LN pathogenesis) show no such effect [50, 69]. HCQ also inhibits other proinflammatory mediators. An in vitro study shows that CQ suppresses both transcriptional activity and steady-state mRNA levels of IL-6 and IL-1β following LPS stimulation [70]. As a hydrophilic analog, HCQ shows comparable or greater potency in suppressing proinflammatory cytokines at both protein and mRNA levels. Additionally, HCQ suppresses TNF-α release, further underscoring its broad anti-inflammatory properties [70].

Activation of TLR9/7 signaling pathways induces mesangial proliferation with significant IgA/IgG/C3 deposition in murine models, which is reversed by HCQ treatment [50, 51]. This protective effect likely stems from HCQ-mediated inhibition of TLR9/7 signaling and subsequent suppression of proinflammatory cytokines (IL-6, IL-1β, TNF-α), ultimately attenuating renal inflammation and proteinuria in IgAN.

#### BAFF/APRIL regulation

In IgAN pathogenesis, BAFF and APRIL drive T-cell-independent IgA CSR. Clinical observations reveal elevated APRIL and BAFF levels in IgAN patients, with BAFF concentrations correlating with IgA levels and renal pathology severity [71]. Murine studies demonstrate that BAFF overexpression induces IgAN-like pathology under commensal flora conditions, accompanied by substantial albuminuria [71]. Notably, clinical trials report BAFF reduction in SLE patients following HCQ treatment [72, 73]potentially through lysosomal alkalinization-mediated inhibition of type I IFN signaling and subsequent BAFF mRNA downregulation [74].

#### Gut barrier protection

HCQ exhibits intestinal protective effects by preserving tight junction proteins (ZO-1, Occludin) and restoring epithelial barrier integrity [10]. This mechanism reduces LPS-TLR4 activation and subsequent Gd-IgA1 production while normalizing gut microbiota composition in IgAN mice. However, HCQ does not directly inhibit TLR4 signaling, as evidenced by its inability to block LPS-stimulated plasmablast differentiation [59].

## HCQ inhibits complement pathway activation

### Complement activation pathways in IgAN

In addition to IgA deposition, renal biopsies from IgAN patients frequently exhibit complement deposition, including C3, C4d, C5b-9, and properdin (P factor) [75]. However, C1q deposition is rare, suggesting that complement activation in IgAN primarily occurs via the alternative and mannan-binding lectin (MBL) pathways. The MBL pathway activates HMCs, triggering the secretion of inflammatory mediators and matrix proteins [76], which exacerbate IgAN progression [77]. A retrospective study of 283 IgAN patients revealed that those with renal C4d deposition exhibited more severe proteinuria, renal dysfunction, and pathological damage than C4d-negative patients [78]. A further study demonstrated that Gd-IgA1 in IgAN patients activates the complement system, promoting circulating IgA1 complex formation [79]. The degree of complement activation correlates strongly with renal injury severity [80, 81]. Additionally, Gd-IgA1 exhibits high affinity for HMCs, stimulating NF-κB expression, which regulates eukaryotic protein kinase phosphorylation, DNA synthesis, and inflammatory factor secretion [82–84]. These processes induce HMC proliferation, inflammatory responses, and intrinsic renal cell injury, ultimately leading to proteinuria in IgAN.

### HCQ as a complement inhibitor in IgAN

Bertolaccini et al. demonstrated that HCQ inhibits the generation of complement split products C3a and C3b/iC3b/C3d in vitro in a mouse model of obstetric antiphospholipid syndrome (APS) [85]. A dose-dependent effect was observed, with complete inhibition at an HCQ concentration of 100 ng/mL. HCQ also abolished C1q binding capacity and C3 activation in treated serum. Compared to β2-glycoprotein I (β2GPI) alone, β2GPI combined with HCQ significantly reduced C3a and C5a levels. Additionally, C5a-desArg levels decreased in APS patients following HCQ treatment [85].

Complement activation plays a critical role in SLE progression to LN [18]. A prospective cohort study revealed that HCQ increases C3 and C4 levels [86]with a particularly strong dose-response relationship between HCQ concentration and C4 elevation. These findings suggest that HCQ may enhance complement levels by reducing IC formation and suppressing complement activation.

**Fig. 3:**
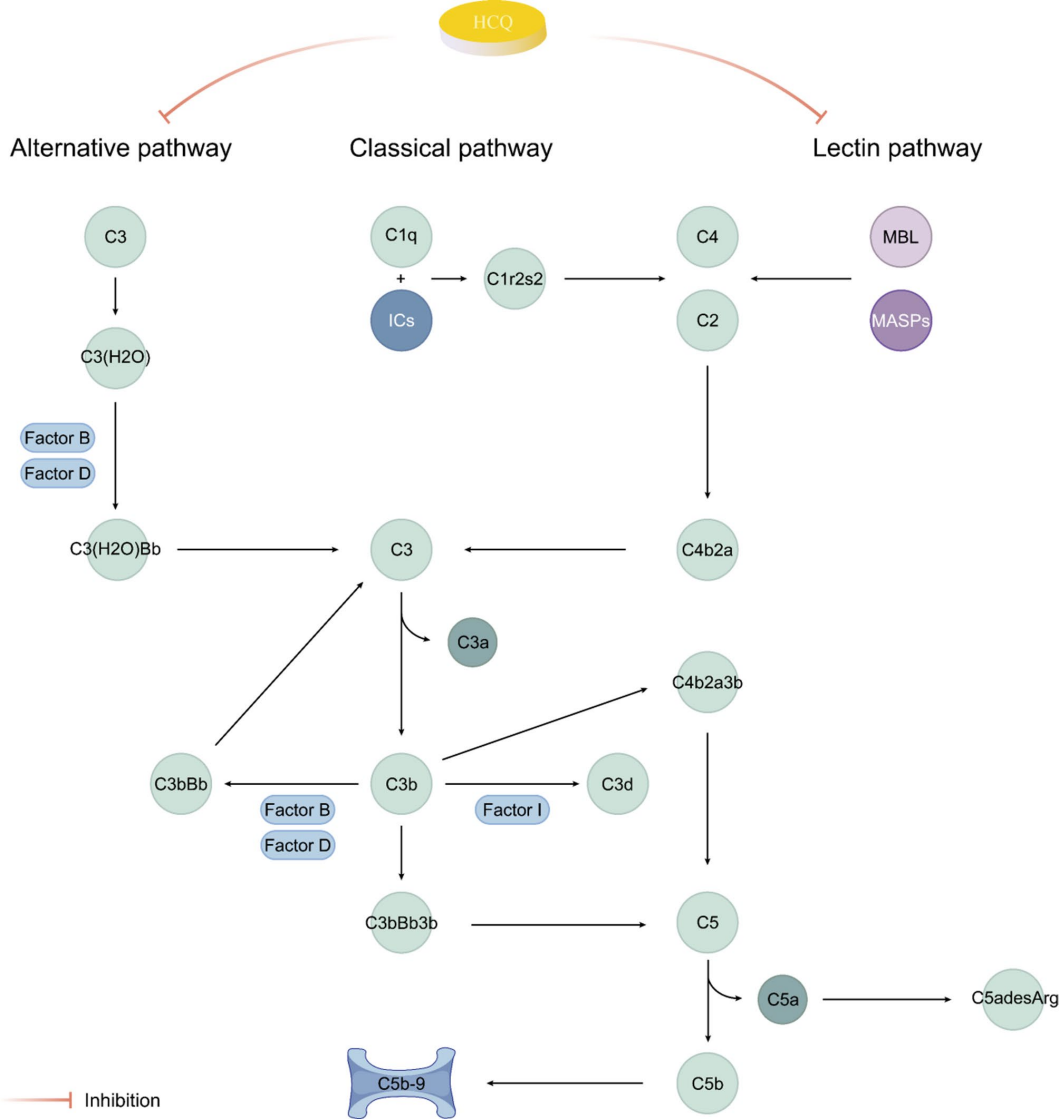
The complement system and the role of HCQ. The complement cascade can be activated via three distinct pathways: the classical pathway, the lectin pathway, and the alternative pathway. The activation of C3 represents the pivotal step in complement activation. In the lectin pathway, the production of acute-phase proteins, such as MBL, bind to microbes, activating MASPs to form C4b2a (a C3 convertase). Conversely, the alternative pathway undergoes continuous low-level self-activation. In the presence of factors B and D, this process leads to the generation of C3b. All three pathways culminate in the formation of C5 convertase (C3bBb3b and C4b2a3b), which cleaves C5. This cleavage releases C5b and the potent anaphylatoxin C5a. C5adesArg is a derivative of C5a. Additionally, C5b initiates the sequential assembly of the lytic membrane attack complex (C5b-9), inducing cell and tissue damage and promoting inflammation. HCQ inhibits both MBL-dependent activation and the alternative pathway. HCQ, hydroxychloroquine; MBL, mannose-binding lectin; MASPs, mannose-binding-lectin-associated serine proteases

Collectively, these studies indicate that HCQ significantly inhibits complement activation, by particularly targeting C3 and C4. C3 serves as a convergence point for all complement activation pathways, while C4 is the key component of the MBL pathway. Given the role of complement in IgAN, we hypothesize that HCQ may ameliorate IgAN and reduce proteinuria by suppressing the MBL and alternative pathways [1] (Fig. 3). However, further investigation is required to elucidate the precise mechanisms.

## HCQ participates in autophagy to relieve IgAN

### HCQ inhibits autophagy and achieves autoimmune regulation

**Fig. 4:**
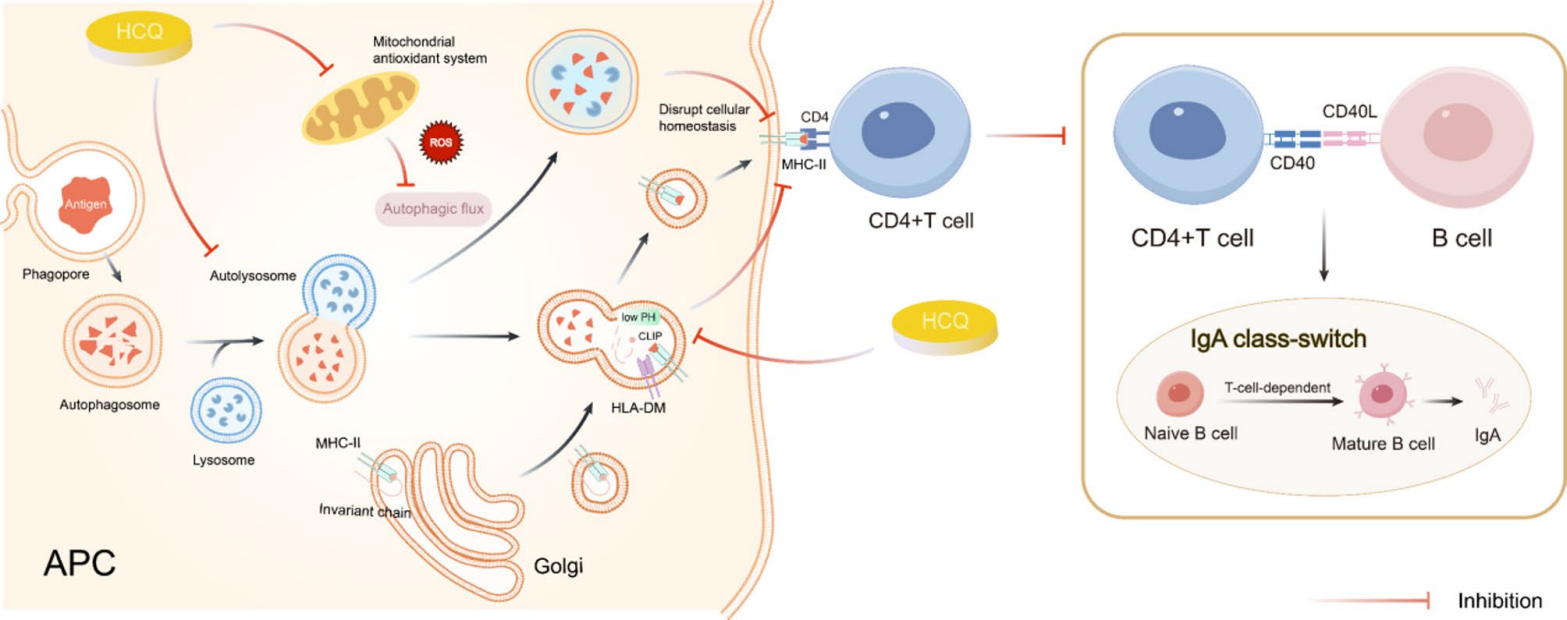
HCQ inhibits autophagy. By alkalizing the lysosome, HCQ disrupts the fusion of autophagosomes with lysosomes. This interference halts autophagic flow and inhibits autophagic flux. Through alkalinization, HCQ impedes post-translational modifications of newly synthesized proteins within the endoplasmic reticulum or trans-Golgi network vesicles. Consequently, HCQ blocks the cleavage of MHC-II invariant chains. Moreover, HCQ suppresses the mitochondrial antioxidant system. This suppression mediates the generation of ROS, leading to excessive ROS production. This inhibits activation-induced autophagic flux, causes autophagosome accumulation, and induces proliferation defects in CD4 + T cells. Cumulatively, these effects inhibit MHC-II autoantigen presentation, thereby suppressing the downstream IgA CRS. HCQ, hydroxychloroquine; MHC-II, major histocompatibility complex class II; ROS, reaction oxidative stress; CRS, class-switch recombination

Autophagy facilitates the presentation of endogenous antigens via MHC-II molecules, with autophagic proteins being essential for CD4 + T cell recognition [87, 88]. HCQ inhibits this pathway through lysosomal alkalinization, which: (1) prevents autophagosome-lysosome fusion [89], (2) disrupts autophagic flux, and (3) impairs MHC-II antigen presentation [90–92] (Fig. 4). By elevating lysosomal pH, HCQ additionally blocks antigen processing and invariant chain cleavage, ultimately suppressing antigen-presenting cell (APC) function. Additionally, HCQ downregulates type I IFN secretion and MHC expression [65].

Kim et al. demonstrated that clinical HCQ concentrations minimally affect MHC-II peptide presentation [64]. Instead, HCQ induces reactive oxygen species (ROS) accumulation, leading to: (1) impaired activation-induced autophagic flux, (2) autophagosome accumulation, and (3) CD4 + T cell proliferation defects. This disrupts CD4 + T-cell cellular homeostasis [93, 94]. Paradoxically, autophagy inhibition in thymic epithelial cells may increase autoreactive T-cell populations [95], suggesting that HCQ does not uniformly suppress MHC-II autoantigen presentation.

Furthermore, HCQ-mediated CD4 + T-cell inhibition reduces CD40L expression, diminishing CD40 binding on B cells and suppressing antibody production [65] (Fig. 4).

HCQ directly inhibits autophagy by upregulating p62 and cleaving caspase-3, blocking autophagy-lysosome fusion [60]. These mechanisms underlie HCQ’s immunomodulatory and anti-inflammatory effects.

### HCQ induces non-canonical autophagy, thereby mitigating renal injury

#### Attenuation of HMCs proliferation

The deposition of Gd-IgA1 ICs activates HMCs, triggering specific intracellular signaling pathways that promote HMCs proliferation and renal injury [33]. Concurrently, stimulated HMCs release pro-inflammatory cytokines and fibroblast factors, which mediate ROS production and complement system activation. These processes ultimately damage podocytes and proximal tubular epithelial cells, manifesting clinically as proteinuria [33, 96, 97]. Wang et al. demonstrated that HCQ treatment in IgAN rat models significantly attenuated HMCs proliferation, reduced inflammatory cell infiltration, and alleviated kidney injury [10].

Emerging evidence indicates that HMCs proliferation in IgAN is autophagy-dependent, playing a pivotal role in disease pathogenesis [98–100]. Liu et al. and Xia et al. reported that autophagy inducers suppress HMCs proliferation via mTOR/S6K1 pathway inhibition in IgAN [98, 99]. Furthermore, IgA-ICs were shown to activate MAPK signaling and increase mitochondrial ROS (mtROS) production, stimulating NLRP3 inflammasome-mediated IL-1β secretion in IC-primed macrophages [101, 102]. Enhanced autophagy inhibits NLRP3 priming and activation, demonstrating therapeutic potential in IgAN mouse models [103].

**Fig. 5:**
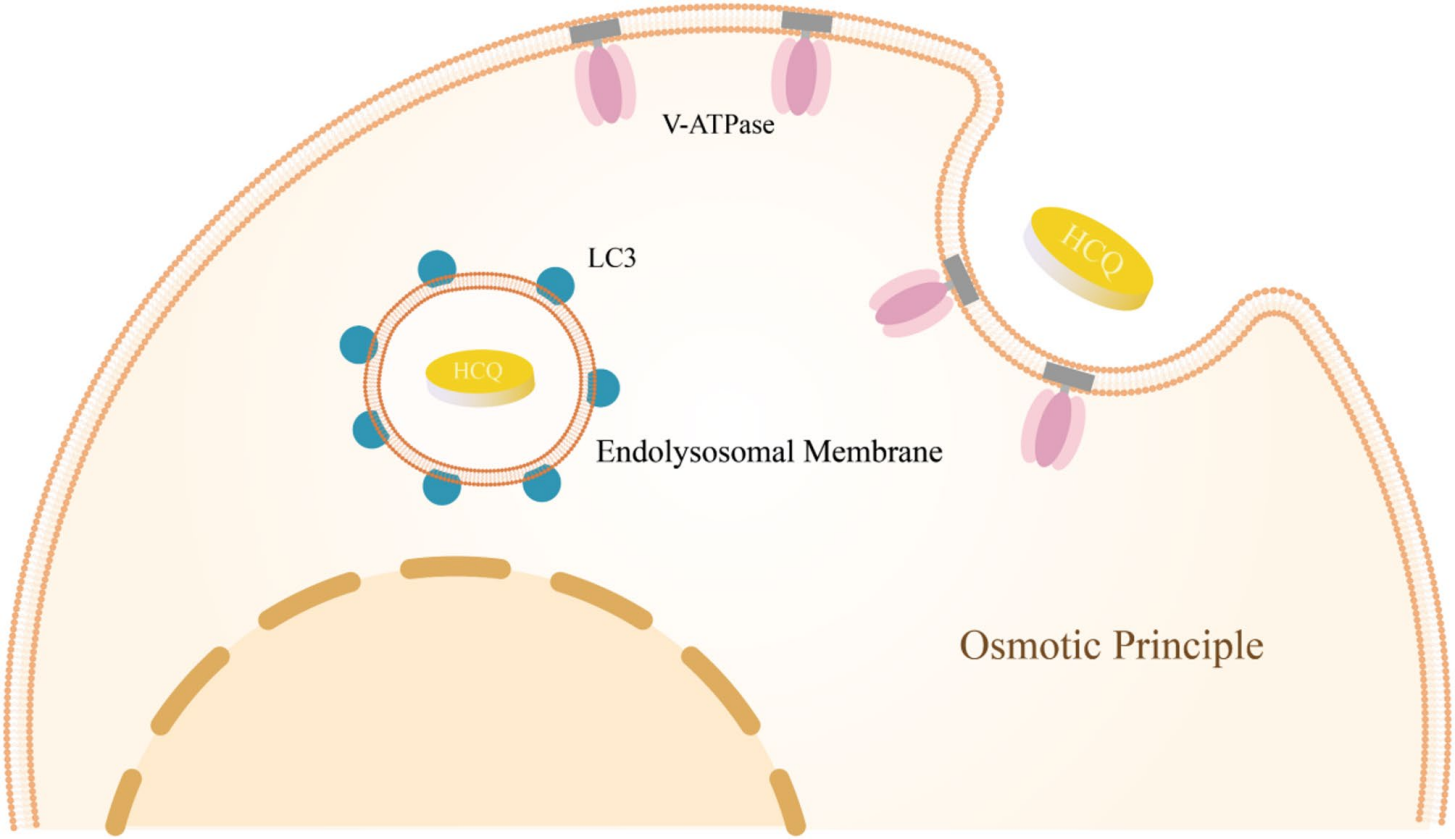
HCQ induces non-canonical autophagy. Facilitated by V-ATPase, HCQ modifies the extracellular tonicity, inducing osmotic imbalance. Subsequently, endocytosis triggers osmotic swelling of endolysosomal compartments, which in turn elicits non-canonical autophagy. V-ATPase, vacuolar-type H^+^-ATPase; HCQ, hydroxychloroquine

Despite being widely characterized as an autophagy inhibitor, CQ and its analog HCQ may induce non-canonical autophagy [94, 104, 105]. This alternative pathway requires vacuolar-type H^+^-ATPase (V-ATPase) activity [104]. Through lysosomotropic action, HCQ alters extracellular tonicity, creating osmotic imbalance that triggers endocytic engulfment events and subsequent endolysosomal compartment swelling [106], ultimately leading to non-canonical autophagy. Intriguingly, while HCQ is a canonical lysosomal autophagy inhibitor, it can simultaneously activate a parallel non-canonical pathway by promoting microtubule-associated protein 1 light chain 3 (LC3) lipidation on endolysosomal membranes [105] (Fig. 5). However, inducing ATG13-independent LC3 lipidation through this mechanism requires higher HCQ concentrations than those needed for canonical autophagy inhibition [105].

#### Alleviation of podocyte injury

The Pl3K-Akt-mTOR pathway is activated in the podocytes of IgAN patients resulting in both suppression of autophagy and injury to podocytes [107]. Severe podocyte injury may result in irreversible glomerular damage [108]. In an in vitro study by Lai et al. demonstrated that culturing podocytes in IgA-conditioned medium prepared from IgAN patients exhibited downregulation of key podocyte proteins (nephrin, ezrin) and markers (podocin, synaptopodin), indicating compromised glomerular filtration barrier integrity [109].

Autophagy induction ameliorated glomerular lesions in IgAN mice [110]. Xue et al. reported that HCQ suppresses inflammatory cytokine production by inhibiting the PI3K-Akt-mTOR pathway [111], while also attenuating epithelial-mesenchymal transition and tubular epithelial cell apoptosis [107]. These mechanisms collectively mitigate renal fibrosis. Additionally, PI3K-Akt-mTOR inhibition may enhance autophagy [112], further limiting fibrotic progression. Network pharmacology analyses by Chen et al. revealed that HCQ negatively regulates PI3K-Akt, AGE-RAGE, and MAPK signaling pathways, suppresses T- and B-cell activation, and reduces atherosclerosis-related gene expression (e.g., RAGE) [107]. Consequently, HCQ alleviates renal vascular sclerosis and inflammatory damage, slowing glomerulonephritis progression and podocyte injury.

## Others

HCQ demonstrates significant antithrombotic properties. Edwards et al. demonstrated that HCQ reduced thrombus size and duration in mice by suppressing tissue factor expression in arterial tissue and lowering plasma levels of soluble E-selectin and vascular cell adhesion molecule-1 (VCAM-1) [113, 114]. HCQ also enhanced endothelial nitric oxide synthase (eNOS) expression, inhibited platelet activation, and promoted vasodilation, supporting endothelial antithrombotic function [113]. Similarly, Gajic et al. observed in preeclampsia patients that HCQ decreased circulating VCAM-1, ICAM-1, and P-selectin, improving endothelial-dependent relaxation and reducing thrombosis [65]. These effects may influence hypertension. Additionally, by reducing endothelial VCAM-1, HCQ may attenuate monocyte infiltration and systemic inflammation [65].

## Limitations

While HCQ shows therapeutic promise in IgAN, several critical limitations must be considered. The current clinical evidence is derived exclusively from Chinese patient cohorts, raising concerns about the generalizability of findings to other ethnic populations. Furthermore, the field suffers from a striking lack of high-quality RCTs with robust designs and adequate power to establish definitive therapeutic efficacy. Preclinical studies face significant translational challenges, as the HCQ dosages employed in animal and cellular models often diverge substantially from clinically relevant regimens, compromising the applicability of these experimental findings to human therapeutics. Perhaps most critically, the precise molecular mechanisms through which HCQ may exert its potential therapeutic effects in IgAN remain incompletely characterized, highlighting the need for more sophisticated mechanistic investigations. These collective limitations underscore both the preliminary nature of current evidence and the necessity for more rigorous, internationally RCTs coupled with detailed mechanistic studies to properly evaluate HCQ’s role in IgAN management.

## Conclusion

Gd-IgA1 drives IgAN pathogenesis. HCQ, repurposed from SLE therapy [16], effectively reduces proteinuria [25] with a safe profile in IgAN [21]. Its mechanisms include: (1) mucosal immunity suppression via TLR9/7, IL-6, and complement inhibition, reducing Gd-IgA1. (2) autophagy modulation, enhancing canonical (MHC-II suppression) and non-canonical (HMC/podocyte protection) pathways (Table 2).

However, robust direct evidence remains limited. Future studies should further elucidate IgAN pathogenesis to optimize treatment strategies. Additionally, long-term clinical trials are needed to evaluate HCQ’s effects on hard clinical endpoints in IgAN, beyond proteinuria reduction alone.

**Table 2:** Summary of the mechanisms by which HCQ improves proteinuria and alleviating IgAN progression

Mitigate mucosal immune response and inhibit Gd-IgA1 synthesis	Inhibit complement pathway activation	Participate in autophagy	Others
• Inhibit the differentiation of memory B cells into plasmablasts to produce. • Suppress TLR9/7 Signaling • Suppress IL-6 secretion • Reduce BAFF/APRIL • Protect gut barrier (Suppress TLR4 signaling indirectly)	• Alternative pathway • Lectin pathway	• Inhibit autophagy Suppress antigen presentation • Induce non-canonical autophagy 1) Attenuate HMCs proliferation 2) Protect podocyte	Antithrombotic properties

HCQ, hydroxychloroquine; IgAN, IgA nephropathy; Gd-IgA1, galactose-deficient IgA1; TLR, Toll-like receptor; BAFF, B cell activation factor; APRIL, a proliferation-inducing ligand; HMCs, human mesangial cells

## Data Availability

No datasets were generated or analysed during the current study.

## Declarations

### Literature search strategy

We have augmented our database searches with Web of Science and PubMed for “IgAN,” “Proteinuria,” “HCQ,” “Gd-IgA1,” “Autophagy,” and related keywords, with a focus on literature published up to 2025. This approach has enabled us to comprehensively track down references to key literature. The literature was initially assessed by title and abstract, followed by a thorough review of the full text to ascertain its relevance. Ultimately, a decision regarding inclusion was made based on the study’s quality, methodological rigor, and its pertinence to the subject matter.
